# Correction: Andritsos et al. Serotyping and Antibiotic Resistance Profiles of *Salmonella* spp. and *Listeria monocytogenes* Strains Isolated from Pet Food and Feed Samples: A One Health Perspective. *Vet. Sci.* 2025, *12*, 844

**DOI:** 10.3390/vetsci13090894

**Published:** 2026-08-31

**Authors:** Nikolaos D. Andritsos, Antonia Mataragka, Nikolaos Tzimotoudis, Anastasia-Spyridoula Chatzopoulou, Maria Kotsikori, John Ikonomopoulos

**Affiliations:** 1 Department of Food Science and Technology, School of Agricultural Sciences, University of Patras, 2 G. Seferi Str., GR-30100 Agrinio, Greece; amataragka@upatras.gr; 2 Laboratory of Anatomy and Physiology of Farm Animals, Department of Animal Science, School of Animal Biosciences, Agricultural University of Athens, 78 Iera Odos Str., GR-11855 Athens, Greece; anactaciahtzp@gmail.com (A.-S.C.); mariakotsikori1620@gmail.com (M.K.); ikonomop@aua.gr (J.I.); 3 Hellenic Army Biological Research Centre, 6 Taxiarchou Velliou Str., P. Penteli, GR-15236 Attica, Greece; n.p.tzimotoudis@army.gr

## Figure Legend

In the original article [1], there was a mistake in the legend for Figure 3. As it is obvious from the photograph referring to the MIC of SXT, strain *L. monocytogenes* BF11 is resistant and not susceptible to this antibiotic due to the absence of any inhibition zone. The correct legend appears below. 

“E-test for minimum inhibitory concentration (MIC) determination of the multidrug resistant *L. monocytogenes* strain BF11 isolated from raw dog food against five antimicrobial agents (antimicrobial class being designated in parenthesis). Antibiotics in yellow and red ultimately mark the respective intermediate resistance and resistance of the BF11 strain against the specific antimicrobial agent, with the conferred resistance due to estimated MIC values > MIC breakpoints provided by EUCAST [33]: (**a**) MIC = 1.5 μg/mL for CIP (fluoroquinolones); (**b**) MIC = 4 μg/mL for MEM (carbapenems); (**c**) MIC = 4 μg/mL for P (penicillins), (**d**) MIC > 1024 μg/mL for SXT (miscellaneous agents), (**e**) MIC = 48 μg/mL for TE (tetracyclines). CIP: ciprofloxacin, MEM: meropenem, P: penicillin, SXT: trimethoprim-sulfamethoxazole, TE: tetracycline, EUCAST: European Committee on Antimicrobial Susceptibility Testing.”

## Error in Figure

In the original article, there was a mistake in Figure 3 as published. SXT antibiotic was marked in green (susceptibility) instead of red (resistance), based on the previous comment provided in the Figure Legend. The corrected Figure 3 appears below. The authors apologize for any inconvenience caused and state that the scientific conclusions are unaffected. The original article has been updated.

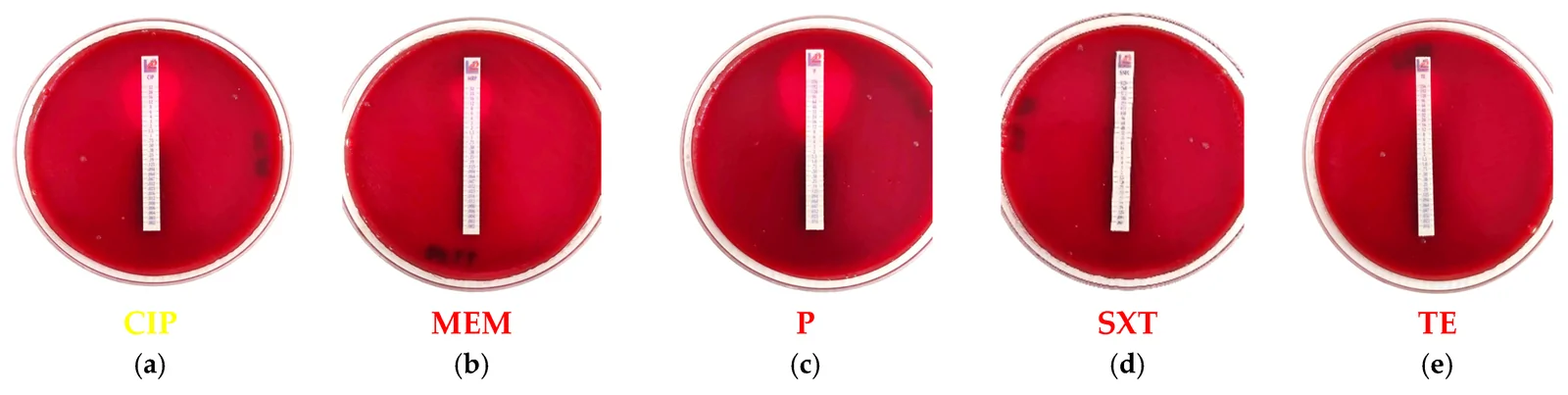



## Text Correction

There was an error in the original article. Because of the aforementioned alteration in the susceptibility/resistance profile of *L. monocytogenes* strain BF11, as depicted by the corrections in Figure 3 and its legend, the following corrections in the original text are necessary.

A correction has been made to the Abstract:

“Foodborne pathogenic bacteria, like *Salmonella* spp. and *Listeria monocytogenes*, can be detected in the primary food production environment. On the other hand, and in the current context of One Health, antimicrobial resistance (AMR) is gaining increased attention worldwide, as it poses significant threat to public health. The purpose of this study was to confirm the presence of *Salmonella* spp. and *L. monocytogenes* in pet food and feed samples, by means of biochemical and/or serological testing of the microbial isolates, and then to screen for AMR against a panel of selected antibiotics. Serotyping of the isolates with multiplex polymerase chain reaction revealed the presence of three of the most common clinical *Salmonella* serovars (*S.* Enteritidis, *S.* Typhimurium, *S.* Thompson) and the major epidemiologically important *L. monocytogenes* serotypes (1/2a, 1/2b, 1/2c, 4b) in 15 and 9 confirmed isolates of the pathogens, respectively. Strains of *Salmonella* spp. showed resistance to tetracycline (*n* = 3) and combined AMR to tetracycline with either ampicillin (*n* = 2) or trimethoprim-sulfamethoxazole (*n* = 3), without any multidrug resistance (MDR) being recorded whatsoever. AMR in *L. monocytogenes* was documented in 55.5% of the bacterial strains (*n* = 5) tested against ciprofloxacin, meropenem, penicillin, trimethoprim-sulfamethoxazole, and tetracycline. Alarmingly, one strain of *L. monocytogenes* was MDR to the latter five antibiotics and deemed resistant in four antibiotic groups (carbapenems, penicillins, sulfonamides, tetracyclines), after exhibiting minimum inhibitory concentrations (MICs) to meropenem (MIC = 4 μg/mL), penicillin (MIC = 4 μg/mL), trimethoprim-sulfamethoxazole (MIC > 1024 μg/mL), and tetracycline (MIC = 48 μg/mL). To the best of our knowledge, finding an MDR *L. monocytogenes* in pet food is something reported for the first time herein. The results presented in this study highlight the presence of important foodborne bacterial pathogens, such as *Salmonella* spp. and *L. monocytogenes*, with increased AMR to antibiotics and possible MDR at the primary production and at the farm level, due to the misuse of pharmacological substances used to treat zoonotic diseases, probably resulting in detection of resistant strains of these pathogenic bacteria in animal-originated food products (e.g., meat, milk, eggs).”

A correction has been made to the Results Section, 3.3. Antibiotic Resistance Profiles of the Pet Food and Feed Bacterial Isolates, paragraph 3:

“MICs for *Salmonella* strains isolated from pet food and feed, which presented resistance through AST, ranged from 2 to 1024 μg/mL, whereas resistance was finally recorded against one (TE) and two antibiotics (i.e., TE and AMP, or TE and SXT) for three and five out of the eight *Salmonella* isolates, respectively (Figure 2a). Additionally, MIC values estimated for the highly resistant *L. monocytogenes* strains isolated from pet food and feed ranged from 0.5 to 1024 μg/mL, whereas all five strains (str. AAL 20148, AAL 20850, AAL 21180, BF9, BF11) were finally deemed resistant to SXT (MIC ≥ 0.5 μg/mL). The MDR *L. monocytogenes* strain BF11 ultimately demonstrated resistance against MEM, P, SXT and TE, with recorded MIC values of 4, 4, >1024 and 48 μg/mL, respectively (Figure 3).”

A correction has been made to the Discussion, paragraph 4:

“The trend of feeding pet dogs and cats raw meat (BARF) and animal by-products because of suggested health benefits to pets as a diet more closely related to their wild-living nature has been linked with the risk of introducing antimicrobial-resistant pathogenic bacteria not only to the raw-fed pets [43,54,66–69] but also in the home food production environment [54,70]. In this study, AST results revealed notable resistance patterns among the bacterial isolates from pet food and feed. Over half of *Salmonella* isolates (53.3%) exhibited resistance to TE, while a subset of these strains (five out of eight) showed resistance to an additional antibiotic (AMP or SXT), though they remained uniformly sensitive to all other tested antimicrobial agents (AMC, CAZ, CIP, CN, CTX, FOX) (Figure 2a). Bacci et al. [67] and Fathi et al. [71] noticed similar resistance patterns to AMP and SXT. The antibiotic resistance profiles of *Salmonella* isolates obtained in this work suggest that while some frontline antibiotics commonly used to treat *Salmonella* infections (e.g., AMC and CIP) [72] remained effective, there is an emerging resistance to widely used antimicrobial agents like tetracyclines [73–77], which are extensively administered as pharmacological substances to treat zoonotic diseases in primary food production at the farm level [19,78,79]. Such an extensive and non-prudent use of antibiotics often leads to the occurrence of residues in food commodities [80,81]. Besides, increased AMR against SXT was observed also in more than half of *L. monocytogenes* isolates (55.6%). Implications for resistance of *L. monocytogenes* against fluoroquinolones (tested with CIP; Figure 2b) suggests that EUCAST correctly points out, in the breakpoint tables provided for the pathogen, that there is insufficient evidence that the organism is a good target for therapy with this specific antibiotic group [34]. Thankfully, all *L. monocytogenes* isolates were susceptible to E and AMP, which are the drugs of choice for treatment of listeriosis [82]. Finally, resistance of the MDR *L. monocytogenes* strain BF11 originally to five and then to four antimicrobial agents, following AST and MIC determination (Figures 2b and 3), respectively, with each agent corresponding to a different antimicrobial class, further emphasizes the difficulties in antibiotic therapeutic schemes followed for treatment of listeriosis. *L. monocytogenes* strain BF11 was finally deemed resistant against penicillin, tetracyclines, sulfonamides and carbapenems (tested with MEM; see Figure 3). Finding MDR *L. monocytogenes* in pet food is something unusual and less commonly reported than MDR *Salmonella* [55]. In fact, this is something reported herein for the first time, to the best of our knowledge, which implies better adaptation of *L. monocytogenes * to environmental stresses and inferred development of cross-resistance, possibly assisting in the wider dissemination of the pathogen in the food chain. This finding also provides an important basis for risk early warning of drug-resistant pathogens transmitted through pet food under the “One Health” framework. Resistance of this particular *L. monocytogenes* strain (BF11) to the antibiotic group of carbapenems is alarming, since carbapenem antibiotics and MEM in particular are considered the last line of defense antimicrobial agents against AMR and MDR bacteria. Especially MEM is useful in treating *L. monocytogenes* meningitis when AMP seems ineffective or if the patient is allergic to AMP [83].”

A correction has been made to Materials and Methods, 2.1. Sampling, Pathogen Isolation, and Confirmation of the Isolates, Paragraph 1:

“Samples of commercial pet food (BARF, i.e., biologically appropriate raw food for dogs and cats), animal feed (fodder in the form of compressed and pelleted feeds, e.g., poultry feed), and a raw feed ingredient (chicken) were collected from January 2015 until May 2022 and microbiologically analyzed for the detection of *Salmonella* spp. and *L. monocytogenes* (Supplementary Tables S1 and S2). Sampling and microbiological analysis were conducted by Eurofins Athens Analysis Laboratories and the Hellenic Army Biological Research Centre. ISO 6579 and ISO 11290-1 protocols were used for the detection of *Salmonella* spp. [24,25] and *L. monocytogenes* [26] from the samples, respectively, as described elsewhere [27]. Briefly, *Salmonella* detection included the overnight pre-enrichment and final enrichment of the initial suspension of the sample in buffered peptone water (Merck, Darmstadt, Germany) and Rappaport-Vassiliadis broth (Merck) at 30 °C and 41.5 °C, respectively, followed by streaking in duplicate onto xylose lysine deoxycholate (XLD) agar (Merck). Detection of *L. monocytogenes* was carried out after primary and secondary enrichment in half-Fraser (BioKar Diagnostics, Pantin, France) and full concentration Fraser broth (BioKar Diagnostics) at 30 °C for 24 h and at 37 °C for 48 h, respectively, followed by streaking in duplicate onto COMPASS^®^ *Listeria* agar (Biokar Diagnostics). Suspect colonies of *Salmonella* on xylose lysine deoxycholate (XLD) agar (Merck) (i.e., black colonies with reddish transparent zone around them) were subcultured on nutrient agar (LabM, Lancashire, UK) for biochemical and serological confirmation of the isolates. Biochemical testing of the isolates took place by performing utilization of triple sugar iron (TSI) agar (Merck), urea agar (Merck), and L-lysine decarboxylation medium (LDC; Merck) [24,25], while it was also achieved by assigning isolates to the genus *Salmonella* using in parallel the RapID™ ONE System (Remel Inc., Thermo Fisher Scientific, San Diego, CA, USA) [28]. Isolates showing typical biochemical reactions for *Salmonella* spp. were subsequently serologically tested with a polyvalent slide agglutination test comprising of poly A-S+Vi and poly H antisera (Statens Serum Institute, Copenhagen, Denmark), for the presence of *Salmonella* O-antigen (somatic), H-antigen (flagellar), and Vi-antigen (capsular). Strains that were confirmed as *Salmonella* spp., showing typical biochemical reactions and positive/negative serological reactions to all or some of the antigens [25], were further typed to serovar level.”

The authors state that the scientific conclusions are unaffected. This correction was approved by the Academic Editor. The original publication has also been updated.
