# Supplementary material for: Serotyping and Antibiotic Resistance Profiles of Salmonella spp. and Listeria monocytogenes Strains Isolated from Pet Food and Feed Samples: A One Health Perspective
Source: Vet Sci. 2025 Sep 1;12(9):844. doi: 10.3390/vetsci12090844 (PMC12474425; doi:10.3390/vetsci12090844)
Supplement: Supplementary file 1 [file vetsci-12-00844-s001.zip › vetsci-3769933-supplementary.pdf]

veterinary sciences

Article

Serotyping and Antibiotic Resistance Profiles of *Salmonella* spp. and *Listeria monocytogenes* Strains Isolated from Pet Food and Feed Samples: A One Health Perspective †

Nikolaos D. Andritsos [1,*], Antonia Mataragka [1,2], Nikolaos Tzimotoudis [3], Anastasia-Spyridoula Chatzopoulou [2], Maria Kotsikori [2] and John Ikonomopoulos [2]

1 Department of Food Science and Technology, School of Agricultural Sciences, University of Patras, 2 G. Seferi Str., GR-30100 Agrinio, Greece
2 Laboratory of Anatomy and Physiology of Farm Animals, Department of Animal Science, School of Animal Biosciences, Agricultural University of Athens, 78 Iera Odos Str., GR-11855 Athens, Greece
3 Hellenic Army Biological Research Centre, 6 Taxiarchou Velliou Str., P. Penteli, GR-15236 Attica, Greece
* Correspondence: nandritsos@upatras.gr; Tel.: +30 26410 74176

† This paper is an extended version of the conference paper: Mataragka, A.; Tzimotoudis, N.; Ikonomopoulos, J.; Andritsos, N.D. Phenotypic characterization and serotyping of *Salmonella* spp. and *Listeria monocytogenes* isolates from feed samples. In Proceedings of the 4th International Electronic Conference on Antibiotics, Online, 21–23 May 2025.

Supplementary Material

Table S1. *Salmonella* spp. isolates from pet food and feed samples used in the study.

Strain No.	Code	Serovar	Strain characterization	Sample	Sampling date	Triple Sugar Iron agar test	Urea test	L-Lysine utilization	Poly A-S+Vi (Poly-O)	Poly-H
1	AAL[1] 10003	Thompson	*S.* Thompson	Poultry feed[2]	27.01.2015	R/Y/+/+[3]	-[4]	+[5]	+	+
2	AAL 10004	Thompson	*S.* Thompson	Poultry feed	27.01.2015	R/Y/+/+	-	+	+	+
3	AAL 10020	Not Determined	*Salmonella* spp.	Poultry feed	24.03.2016	R/Y/+/+	-	+	+	+
4	AAL 10022	Not Determined	*Salmonella* spp.	Poultry feed	01.04.2016	R/Y/+/+	-	+	+	+
5	AAL 10023	Not Determined	*Salmonella* spp.	Poultry feed	01.04.2016	R/Y/+/+	-	+	+	+
6	AAL 10048	Enteritidis	*S.* Enteritidis	Pet food (BARF[6]) with chicken meat	29.09.2016	R/Y/+/+	-	+	+	+
7	AAL 10068	Thompson	*S.* Thompson	Animal feed[2]	08.11.2016	R/Y/+/+	-	+	+	+

8	AAL 10077	Not Determined	*Salmonella* spp.	Animal feed	21.11.2016	R/Y/+/+	-	+	+	+
9	AAL 10201	Thompson	*S.* Thompson	Pet food (BARF) with chicken meat	18.09.2018	R/Y/+/+	-	+	+	+
10	AAL 10202	Thompson	*S.* Thompson	Pet food (BARF) with chicken meat	18.09.2018	R/Y/+/+	-	+	+	+
11	AAL 10228	Thompson	*S.* Thompson	Pet food (BARF) with beef meat	20.02.2019	R/Y/+/+	-	+	+	+
12	AAL 10229	Typhimurium	*S.* Typhimurium	Pet food (BARF) with chicken meat	21.02.2019	R/Y/+/+	-	+	+	+
13	AAL 10646	Thompson	*S.* Thompson	Raw feed ingredient (chicken)	20.04.2021	R/Y/+/+	-	+	+	+
14	AAL 10647	Thompson	*S.* Thompson	Raw feed ingredient (chicken)	20.04.2021	R/Y/+/+	-	+	+	+
15	AAL 10658	Thompson	*S.* Thompson	Pet food (BARF) with pork meat	13.05.2021	R/Y/+/+	-	+	+	+

[1] AAL: Eurofins Athens Analysis Laboratories; Microbiology Laboratory, Metamorfosi, Attica, Greece.

[2] Fodder in the form of compressed and pelleted feeds.

[3] R/Y/+/+: Red slant/Yellow butt/Gas production/H_2S produced

[4] -: negative reaction/result.

[5] +: positive reaction/result.

[6] BARF: Biologically Appropriate Raw Food (for dogs and cats).

Table S2. *Listeria monocytogenes* isolates from pet food and feed samples used in the study.

Strain No.	Code	PCR-serogroup[1]	Serotype[1]	Sample	Sampling date	Catalase reaction	Oxidase test	Motility at 25 °C	Hemolysis on sheep blood agar	CAMP test	L-Rhamnose utilization	D-Xylose utilization
1	AAL[2] 20148	IVb	4b, 4d, 4e	Poultry feed[3]	02.11.2016	+[4]	-[5]	+	+	SA+/RE-[6]	+	-
2	AAL 20849	IVb	4b, 4d, 4e	Pet food (BARF) with chicken meat	06.12.2019	+	-	+	+	SA+/RE-	+	-
3	AAL 20850	IIa	1/2a, 3a	Pet food (BARF) with chicken meat	06.12.2019	+	-	+	+	SA+/RE-	+	-
4	AAL 20860	IVb	4b, 4d, 4e	Pet food (BARF) with chicken meat	18.12.2019	+	-	+	+	SA+/RE-	+	-
5	AAL 21180	IIa	1/2a, 3a	Animal feed	18.02.2021	+	-	+	+	SA+/RE-	+	-
6	BF[7]3	IIa	1/2a, 3a	Pet food (BARF)	11.04.2022	+	-	+	+	SA+/RE-	+	-
7	BF9	IIc	1/2c, 3c	Pet food (BARF)	26.05.2022	+	-	+	+	SA+/RE-	+	-
8	BF10	IIa	1/2a, 3a	Pet food (BARF)	26.05.2022	+	-	+	+	SA+/RE-	+	-
9	BF11	IIb	1/2b, 3b	Pet food (BARF)	26.05.2022	+	-	+	+	SA+/RE-	+	-

[1] PCR-serogroups and serotypes of *L. monocytogenes* were assigned by mPCR using the protocol of Doumith et al. [30].

[2] AAL: Eurofins Athens Analysis Laboratories; Microbiology Laboratory, Metamorfosi, Attica, Greece.

[3] Fodder in the form of compressed and pelleted feeds.[4] +: positive reaction/result.

[5] -: negative reaction/result.

[6] SA+/RE-: positive reaction with *Staphylococcus aureus* and negative reaction with *Rhodococcus equi* (CAMP test).

[7] BF strains were kindly provided by the Hellenic Army Biological Research Centre, Microbiology Laboratory, P. Penteli, Attica, Greece.

Table S3. *Salmonella* spp. isolates (n = 15) from pet food and feed samples used in the study per antibiotic tested.

Strain No.	Code	Strain characterization[1]	Antibiotic									Antimicrobial resistance per sample
			AMC	AMP	CAZ	CIP	CN	CTX	FOX	SXT	TE	
1	AAL[2] 10003	*S*. Thompson	S[3]	S	S	S	S	S	S	S	S	0
2	AAL 10004	*S*. Thompson	S	S	S	S	S	S	S	S	S	0
3	AAL 10020	*Salmonella* spp.	S	S	S	S	S	S	S	S	S	0
4	AAL 10022	*Salmonella* spp.	S	S	S	S	S	S	S	S	S	0
5	AAL 10023	*Salmonella* spp.	S	S	S	S	S	S	S	S	S	0
6	AAL 10048	*S*. Enteritidis	S	S	S	S	S	S	S	R[4]	R	2
7	AAL 10068	*S*. Thompson	S	S	S	S	S	S	S	S	S	0
8	AAL 10077	*Salmonella* spp.	S	S	S	S	S	S	S	R	R	2
9	AAL 10201	*S*. Thompson	S	S	S	S	S	S	S	S	R	1

10	AAL 10202	*S*. Thompson	S	S	S	S	S	S	S	S	R	1
11	AAL 10228	*S*. Thompson	S	S	S	S	S	S	S	S	R	1
12	AAL 10229	*S*. Typhimurium	S	S	S	S	S	S	S	S	S	0
13	AAL 10646	*S*. Thompson	S	R	S	S	S	S	S	S	R	2
14	AAL 10647	*S*. Thompson	S	R	S	S	S	S	S	S	R	2
15	AAL 10658	*S*. Thompson	S	S	S	S	S	S	S	R	R	2

AMC: amoxicillin-clavulanate, AMP: ampicillin, CAZ: ceftazidime, CIP: ciprofloxacin, CN: gentamicin, CTX: cefotaxime, FOX: cefoxitin, SXT: trimethoprim-sulfamethoxazole, TE: tetracycline.

[1] Serotypes of L. monocytogenes were assigned by mPCR using the protocol of Doumith et al. [30].

[2] AAL: Eurofins Athens Analysis Laboratories; Microbiology Laboratory, Metamorfosi, Attica, Greece.

[3] S: Sensitive.

[4] R: Resistant.

Table S4. *L. monocytogenes* isolates (n = 9) from pet food and feed samples used in the study per antibiotic tested

Strain No.	Code	Serotype characterization	Antibiotic							Antimicrobial resistance per sample
			AMP	CIP	E	MEM	P	SXT	TE	
1	AAL[1] 20148	4b, 4d, 4e	S[2]	R[3]	S	S	S	R	S	2
2	AAL 20849	4b, 4d, 4e	S	I[4]	S	S	S	S	S	0
3	AAL 20850	1/2a, 3a	S	I	S	S	S	R	S	1
4	AAL 20860	4b, 4d, 4e	S	I	S	S	S	S	S	0
5	AAL 21180	1/2a, 3a	S	I	S	S	S	R	S	1
6	BF[2]3	1/2a, 3a	S	I	S	S	S	S	S	0
7	BF9	1/2c, 3c	S	I	S	S	S	R	S	1
8	BF10	1/2a, 3a	S	I	S	S	S	S	S	0
9	BF11	1/2b, 3b	S	R	S	R	R	R	R	5

AMP: ampicillin, CIP: ciprofloxacin, E: erythromycin, MEM: meropenem, P: penicillin, SXT: trimethoprim-sulfamethoxazole, TE: tetracycline.

[1] AAL: Eurofins Athens Analysis Laboratories; Microbiology Laboratory, Metamorfosi, Attica, Greece.

[2] S: Sensitive.

[3] R: Resistant.

[4] I: Intermediate.
